# When Life-Threatening Injuries Overshadow Soft Tissue Trauma: A Missed Morel-Lavallée Lesion

**DOI:** 10.7759/cureus.104806

**Published:** 2026-03-07

**Authors:** James C Montgomery, Holden Lewis, Henry Knox, Carolyn Dudko, Saptarshi Biswas

**Affiliations:** 1 General Surgery, Grand Strand Healthcare System, Myrtle Beach, USA; 2 Medicine, Edward Via College of Osteopathic Medicine-Carolinas Campus, Spartanburg, USA; 3 Trauma and Acute Care Surgery, Grand Strand Regional Medical Center, Myrtle Beach, USA

**Keywords:** delayed diagnosis, high-energy trauma, morel-lavallée lesion, polytrauma, soft tissue injury

## Abstract

Morel-Lavallée lesions are closed, shearing injuries that occur in polytrauma patients, most commonly affecting the thigh, hip, and pelvis due to mobile skin over thick fascia. These lesions are often missed upon initial evaluation due to the severity of accompanying traumatic injuries and the focus on life-threatening conditions. We present a case of a missed Morel-Lavallée lesion in a Level 1 trauma patient and our innovative approach to treatment.

A 36-year-old female patient was air-transported to the emergency department after a motorcycle accident, where she was struck by a vehicle. She arrived intubated with a Glasgow Coma Scale of 3, sustaining a skull fracture and multiple injuries requiring exploratory laparotomy and fracture fixation.

Amidst these critical injuries, a Morel-Lavallée lesion in the right anterior-medial thigh was overlooked. On hospital day 10, the patient developed increasing pain and swelling in her right thigh, prompting further evaluation. A computed tomography (CT) scan revealed the Morel-Lavallée lesion. The lesion, now with an eschar, was managed with serial washouts, debridements, and wound vacuum-assisted therapy. Persistent pain and inflammation necessitated surgical intervention, revealing dermal and subcutaneous defects. Treatment included split-thickness skin grafts, autologous skin harvesting, autografts, and dermal substitute application, followed by vacuum-assisted closure. The patient underwent a final wound washout three weeks later before discharge to rehabilitation.

This case highlights how Morel-Lavallée lesions are easily missed in the acute setting due to the prioritization of life-threatening injuries. Recognizing and managing these lesions is crucial to preventing complications such as infection and skin necrosis. This case provides insight into both operative and non-operative innovative treatment strategies for these complex soft tissue injuries.

## Introduction

Morel-Lavallée lesions are a closed, post-traumatic, internal degloving injury where the skin and subcutaneous tissue are torn away from the underlying fascia due to severe shear forces, typically occurring in patients with polytrauma [1]. These lesions are frequently underdiagnosed at initial presentation due to the presence of distracting fractures or concomitant skin abrasions. If left unrecognized, Morel-Lavallée lesions can result in damage to adjacent tissues and lymphatic structures, leading to significant long-term pain and morbidity [2]. These lesions predominantly affect areas around the proximal femur and pelvis, where the superficial anatomy of the femoral region predisposes to these injuries. The pathophysiology of Morel-Lavallée lesions involves the separation of the subcutaneous tissue from the underlying fascial plane, resulting in the formation of a cavity [3].

Despite their impact, Morel-Lavallée lesions are often missed in the acute trauma setting. The presence of distracting injuries, such as fractures and abrasions, along with their closed nature, makes initial detection challenging. Notably, up to 33% of cases may present with delayed onset, manifesting months to years after the initial trauma [4]. Clinically, these lesions often resemble abscesses, with overlying ecchymosis, anesthesia due to nerve injury, or even necrosis [5]. Their delayed and variable presentation necessitates a high index of suspicion, particularly in patients with trauma involving the proximal thigh and pelvis regions, which are most prone to these injuries.

Although clinical examination remains key for diagnosis, imaging can provide confirmation, especially in atypical cases. Magnetic resonance imaging (MRI) is the most sensitive modality, revealing well-defined fluid collections between fascial planes, often with tapering margins. Understanding the pathophysiology and recognizing the characteristic presentation of Morel-Lavallée lesions are essential to preventing complications and ensuring timely intervention.

## Case presentation

A 36-year-old female patient presented to the emergency department after being thrown off a motorcycle into oncoming traffic and run over by another vehicle. She was intubated in the field for airway protection and airlifted to a Level I Trauma Center. The patient presented in extremis with profound hypotension. The extended focused assessment with sonography in trauma (eFAST) was positive in multiple quadrants with the absence of right-lung sliding. A right chest tube was placed. Subsequently, the massive transfusion protocol (MTP) was initiated in the trauma bay, and the patient was taken to the operating room for urgent exploratory laparotomy. She underwent splenectomy and hepatorrhaphy for a grade V liver injury and received 14 units of packed red blood cells (pRBC), 12 units of fresh frozen plasma (FFP), 12 platelets, and two cryoprecipitate units given over the course of the case. Over her course in the intensive care unit (ICU), she required multiple open reductions and internal fixation of tibia and fibular fractures, medial collateral ligament (MCL)/meniscus repair, chest wall reconstruction along with multiple rib and sternal plating, four-level lumbar fusion, and finally a thoracic endovascular aortic repair (TEVAR).

**Figure 1:**
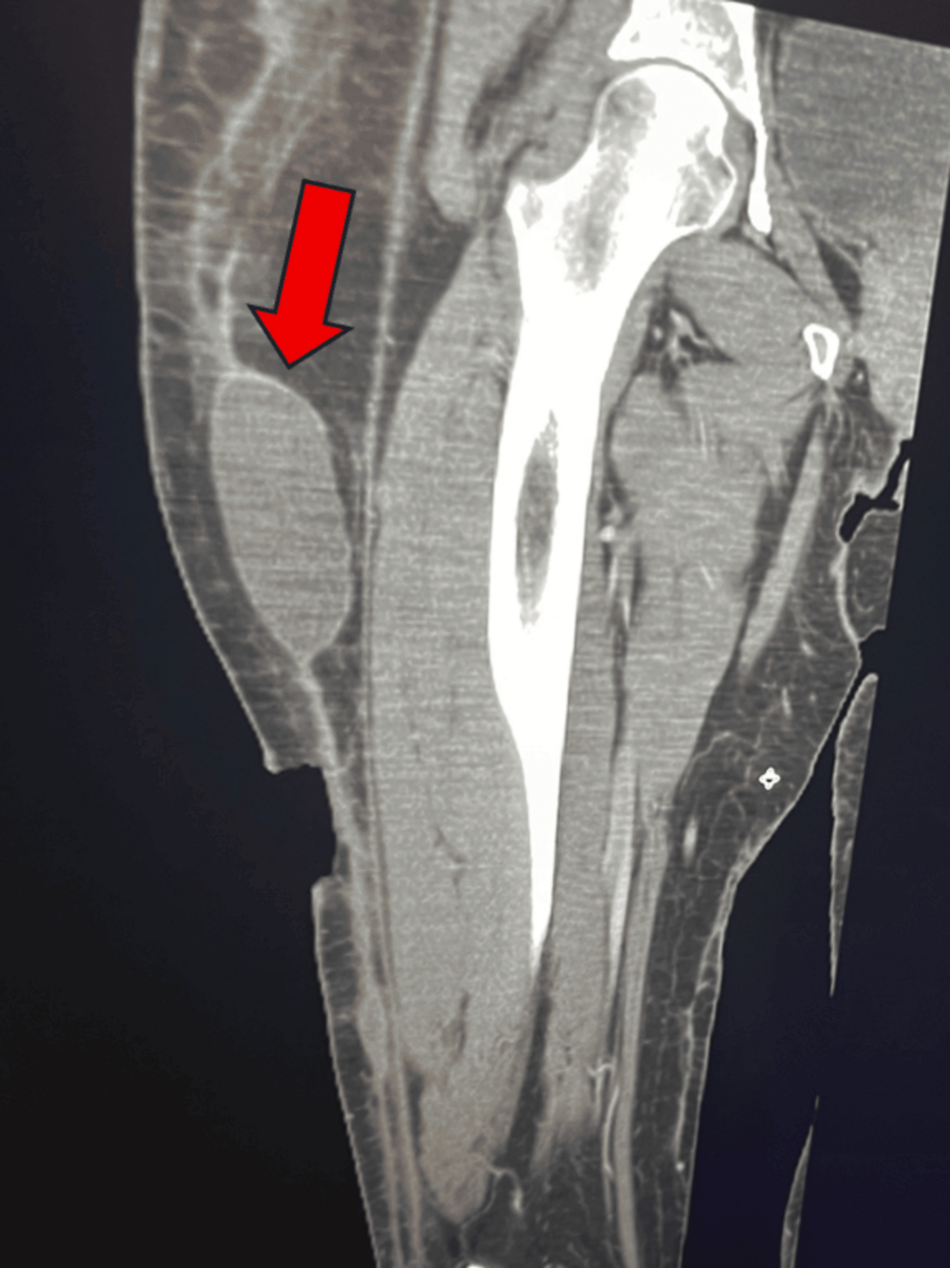
Extensive Subcutaneous Fluid Collection of the Right Anterior Thigh Consistent With Suspected Morel-Lavallée Lesion Axial computed tomography (CT) image of the right thigh demonstrating a large, attenuated fluid collection within the subcutaneous adipose tissue involving the entirety of the anterior thigh (red arrow). Imaging was obtained to evaluate for a suspected Morel-Lavallée lesion and/or underlying abscess following progression of an anterior thigh wound to eschar formation.

On hospital day 9, the patient began to develop pain and swelling in her right anterior thigh (Table 1). The wound on the anterior thigh, which was managed by the wound care team initially, had developed into an eschar. A computed tomography (CT) scan was performed to rule out a Morel-Lavallée lesion and/or underlying abscess cavity. Ensuing imaging indicated a large, attenuated fluid collection within the adipose involving the entirety of the right thigh (Figure 1).

**Table 1:** Clinical Timeline of Hospital Course Following Life-Threatening Motorcycle Collision, Highlighting the Diagnosis and Management of a Right Anterior Thigh Morel-Lavallée Lesion. The patient underwent operative washout and debridement beginning on hospital day 12 after radiographic identification of a fluid collection on day 9, followed by wound dehiscence on day 16, serial debridements (hospital days 17–37), split-thickness skin grafting on day 38, and eventual wound vacuum removal with discharge to rehabilitation on day 44.

Hospital Day (#)	Course of Action
0	Patient airlifted to Level 1 Trauma Center after a life-threatening motorcycle accident. extended focused assessment with sonography in trauma (eFAST) positive in multiple quadrants, absent right lung sliding, right chest tube placed, massive transfusion protocol initiated, urgent exploratory laparotomy with splenectomy and hepatorrhaphy for a grade V liver injury.
1-8	Multiple orthopedic surgeries, including open reduction and internal fixation (ORIF) for tibia and fibular fractures, medial collateral ligament (MCL)/meniscus repair, chest wall reconstruction with multiple rib and sternal plating, four-level lumbar fusion, and a thoracic endovascular aortic repair (TEVAR).
9	Morel-Lavallée lesion suspected. The CT scan showed an attenuated fluid collection involving the right anterior thigh.
12	Taken to the OR for washout and debridement.
14	Wound measured 33cm x 25cm x 2.5cm.
16	Complete dehiscence of the skin layer; wound measured 32cm x 30cm x 2.5cm.
17-37	Serial washouts and debridements to prepare the wound bed.
38	Split-thickness skin graft placed.
44	Wound vac removed and patient discharged to rehab.

**Figure 2:**
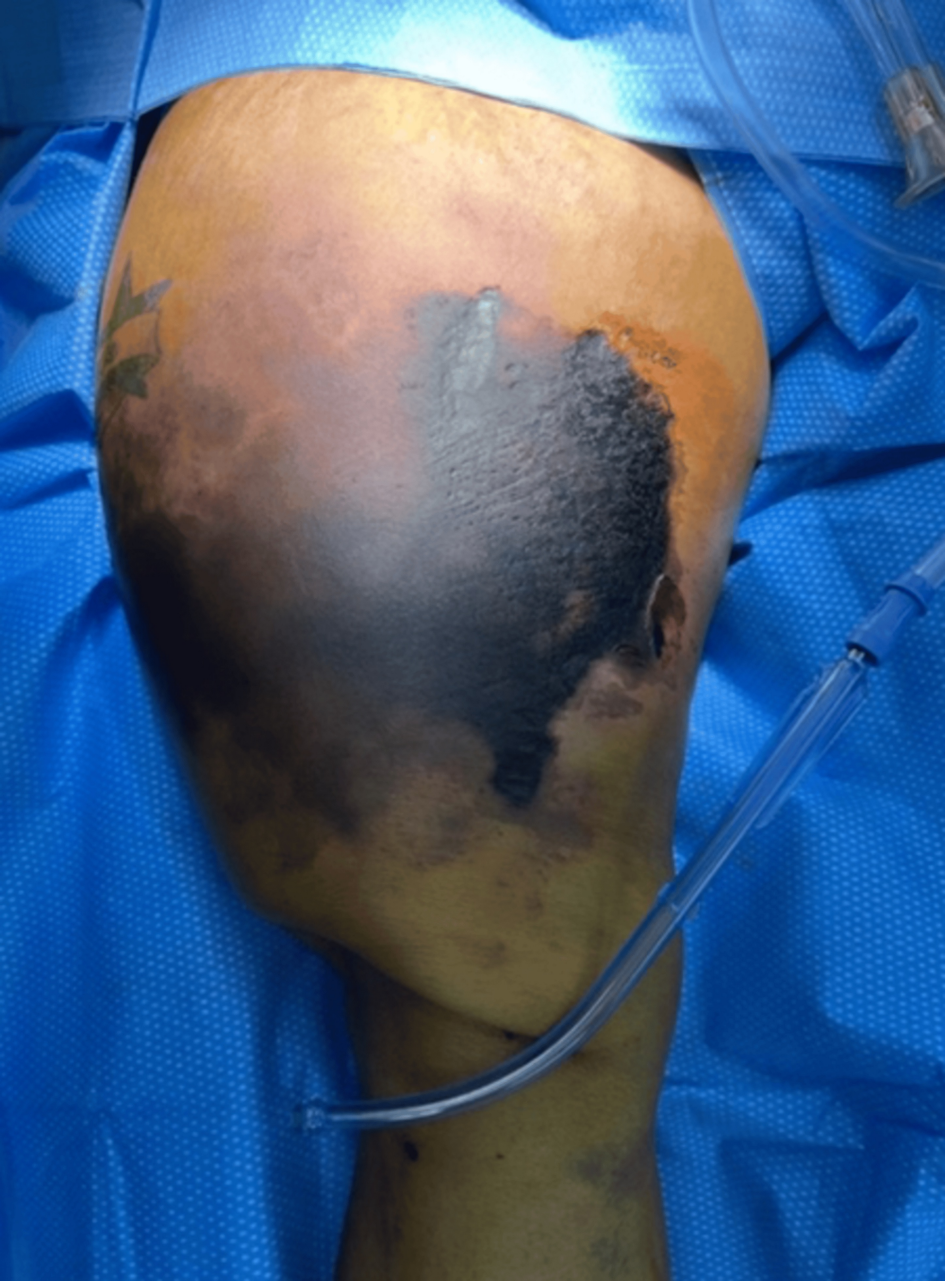
Preoperative Findings Demonstrating Extensive Right Thigh Hematoma Preoperative photograph obtained prior to excisional debridement and hematoma evacuation.

Excisional debridement with hematoma evacuation of her right thigh was scheduled. Intraoperative findings revealed an extensive shearing injury of the thigh from the right inguinal ligament down to the level of the knee, along with a large eschar with full-thickness skin necrosis down to the underlying fat (Figure 2). A hematoma, as well as over a liter of lymphatic fluid, was evacuated from the lesion. The wound was filled with vancomycin powder, which served as a topical antibiotic and a sclerosing agent. Two Penrose drains, along with wet-to-dry dressings, were then placed to facilitate further fluid evacuation. Perioperative systemic antibiotics were administered during each of the multiple trips to the operating room for orthopedic procedures. However, the Morel-Lavallée lesion required topical antibiotics. First, minocycline mixed with the V.A.C. Veraflo Therapy System (3M Company, St. Paul, MN, USA) was administered. Then, vancomycin topical powder was used to prevent deep and superficial infections. Besides being a potent antibiotic, minocycline also acts as a sclerosing agent, helping to keep the seroma down and prepare the tissue bed for a skin graft. Minocycline acts as an irritating agent leading to inflammation of the endothelial linings, fibrosis, clot formation, and lesion involution. In addition, minocycline inhibits matrix metalloproteinases, which reduces angiogenesis and collagen degradation [6, 7]. Source control with excision of eschar/necrotic tissue was performed as staged procedures. The patient returned to the operating room (OR) three days later for further washout and debridement. At this time, the patient was found to have more necrotic tissue, areas of thrombosed vessels, patches of necrotic fat, and complete separation of the skin/subcutaneous layers from the underlying fat and muscle layers. Previous Penrose drains were removed, and the patient underwent further debridement of necrotic tissue. Pulse lavage was used to clean the debris, and a silver wound vacuum was applied over a non-adhering dressing. Orthopedic broad vessel loops using the Roman Sandal technique were used over the vacuum to shrink the wound's dimensions.

**Figure 3:**
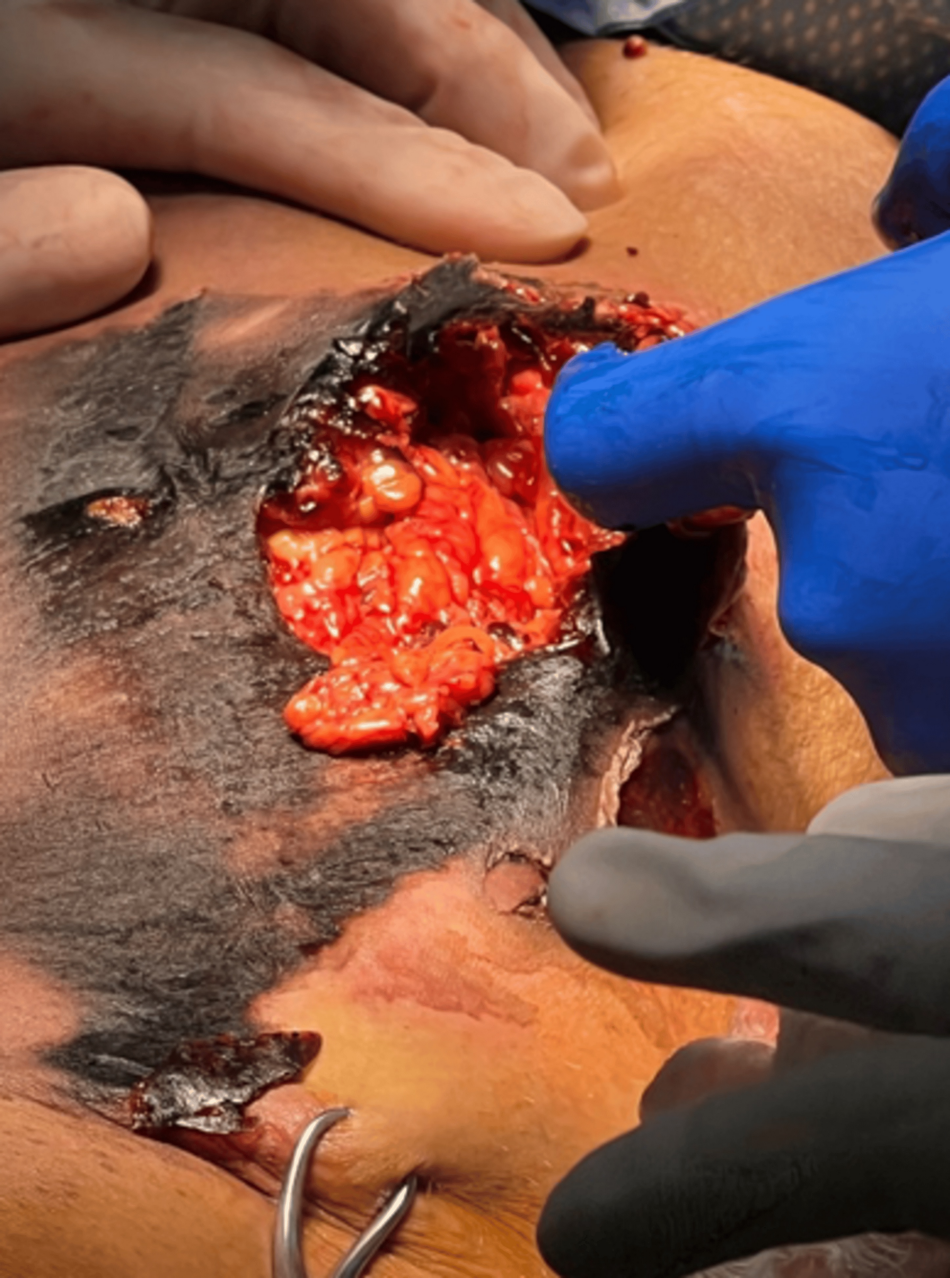
Complete Separation of Anterior Right Thigh Soft Tissue from Underlying Musculofascial Structures Intraoperative image obtained on hospital day 16 demonstrating complete detachment of the anterior right thigh soft tissue envelope from the underlying subcutaneous fat and muscle layers.

Upon her next return to the OR on hospital day 16, the entirety of the anterior right thigh was found to be completely separated from the underlying fat and muscle layers (Figure 3). Three syringes of doxycycline hyclate and lidocaine were applied to the lesion. Subsequently, 3M Veraflo wound vacuum therapy (Solventum, Saint Paul, MN, USA) was applied to the wound, which now measured 32 cm x 30 cm x 2.5 cm compared to 33 cm x 25 cm x 2.5 cm on hospital day 14. Veraflo therapy was started at -100 mmHg with normal saline circulation.

Vicryl interrupted sutures (Ethicon, Raritan, NJ, USA) were placed at the wound edges along with dermal matrices at the lateral and medial portions of the wound to facilitate closure. Fenestrated sheets with powder were placed underneath the negative pressure wound system. Over the course of the next month, the patient required multiple trips to the OR with washouts and debridements before the wound bed was ready with healthy granulation tissue suitable for skin grafting.

**Figure 4:**
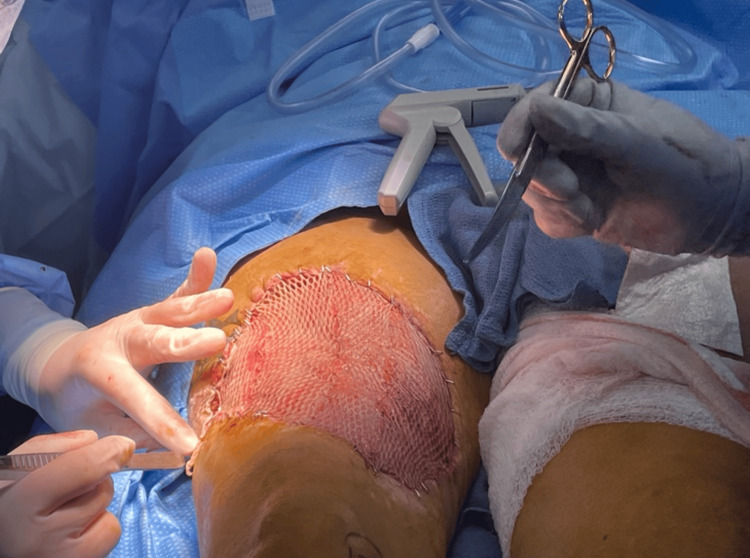
Split-Thickness Skin Grafting of Right Anterior Thigh Following Granulation Intraoperative image obtained approximately one month after the index procedure demonstrating a well-granulated right anterior thigh wound measuring 27 × 16 cm. A 3:1 meshed split-thickness autologous skin graft was applied to the defect.

**Figure 5:**
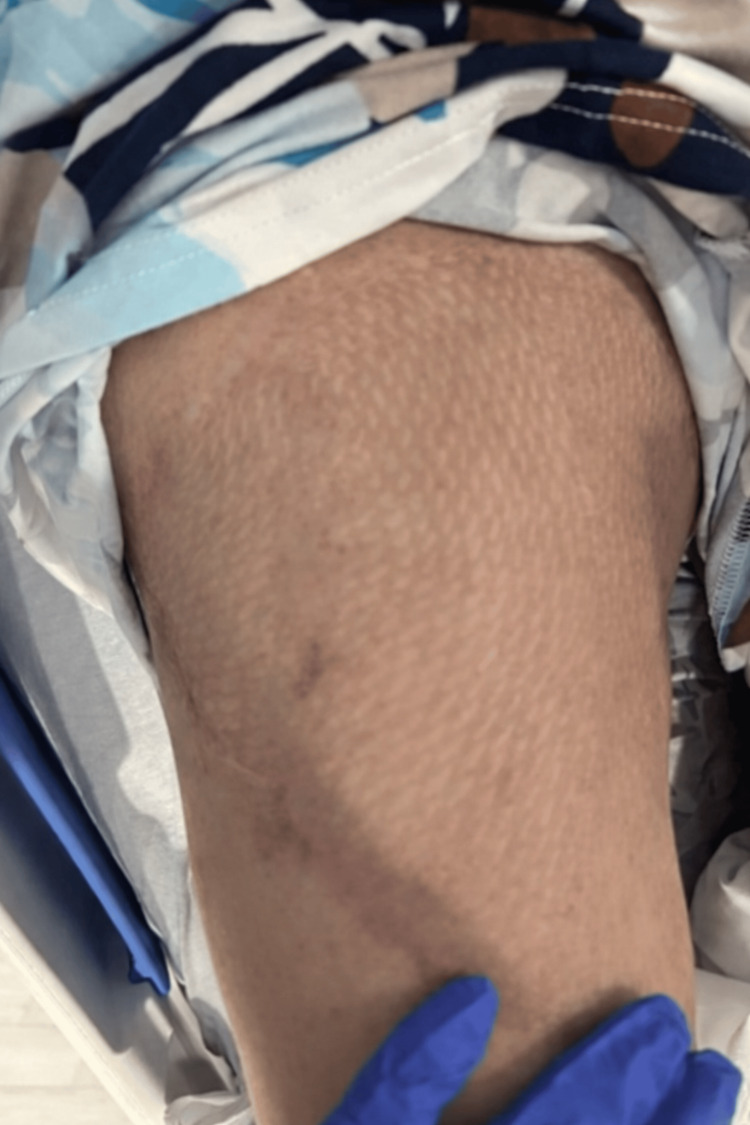
Well-Healed Split-Thickness Skin Graft Following Adjunctive Epidermal Skin Concentrate Therapy Postoperative image demonstrating a well-incorporated and fully epithelialized right anterior thigh split-thickness skin graft after application of epidermal skin concentrate and six days of silver negative pressure wound therapy at −125 mm Hg. The graft demonstrates excellent take with uniform healing, and the patient was subsequently discharged to rehabilitation to continue recovery.

Almost a month from the index case, the patient returned to the OR for planned split-thickness skin grafting. The right anterior thigh wound demonstrated healthy granulation tissue and measured 27 cm x 16 cm. A split-thickness autologous skin graft was meshed 3:1 and applied to the right anterior thigh (Figure 4). Epidermal skin concentrate liquid was then sprayed over the skin graft to minimize the need for a larger donor graft, considering the multiple injuries and scarcity of a suitable donor site. The epidermal skin concentrate was also applied to the left thigh donor site to facilitate regeneration. A silver negative pressure wound therapy was attached to a suction set at 125 mmHg and removed only after six days. The graft was well taken, and the patient was discharged to rehab and continued her remarkable recovery (Figure 5).

## Discussion

Morel-Lavallée lesions are uncommon soft tissue injuries that typically occur in patients who have sustained polytrauma. These lesions are most frequently observed in individuals aged 30 to 40 years [8]. Due to the urgency associated with other concurrent injuries, Morel-Lavallée lesions may be overlooked at initial presentation or exhibit delayed symptoms; some of these lesions have even been reported in the literature 20 years after initial presentation [9]. Early signs are often subtle, and the lesions may not be clinically apparent during the initial assessment. MRI and ultrasound are considered the most effective imaging modalities for diagnosing these lesions. However, a CT scan in a trauma situation is equally effective. These lesions often go unrecognized because they resemble hematomas or contusions [10]. Acute lesions will present as hyperdense without a pseudocapsule, whereas evolving lesions become hypodense and develop a well-marginated pseudocapsule. Thus, a high clinical index of suspicion must be present.

Given the significant recurrence rate associated with Morel-Lavallée lesions, early intervention is crucial. Once identified, drainage or debridement is often required. While there is no universally accepted treatment protocol, management strategies typically range from conservative measures such as bandaging and drainage for smaller lesions to surgical intervention for more extensive lesions. The management of these lesions must be tailored to the extent of soft tissue injury, the presence of necrosis, and the risk of recurrence for each case. In cases involving necrotic tissue or eschar, early surgical debridement may be necessary, which informed our decision to pursue prompt debridement and subsequent skin grafting [11].

In this case, conservative approaches were not viable since the extent of tissue damage was substantial, with full-thickness skin loss and complete separation of the dermal and subdermal planes, necessitating serial washouts and staged debridements to fully demarcate viable from nonviable tissue. Notably, after performing serial debridements, we incorporated autologous skin cell solution in conjunction with the skin graft to accelerate healing and promote tissue regeneration. To minimize the risk of recurrence, we also utilized sclerosing agents, which have been shown to induce cellular destruction and promote fibrosis [12]. The autologous skin cell suspension utilized has shown promise in treating acute, severe skin injuries. The main benefit of this solution is its ability to promote faster re-epithelialization while minimizing the size of the donor site [13].

Given the large surface area of the wound and the patient's limited donor site availability, we opted to utilize an autologous skin cell suspension to expand the coverage of a relatively small split-thickness skin graft. This technology allows for the harvesting of a minimal donor site while creating a sprayable suspension of regenerative epidermal cells, which can be applied over both the graft and the donor site to promote rapid epithelialization. This approach was particularly advantageous in this case, as it minimized additional trauma to the patient while ensuring effective coverage of a complex wound bed. Moreover, by combining this technique with acellular dermal matrix and sclerosing agents, we enhanced tissue integration and reduced the likelihood of fluid reaccumulation or recurrence. This case demonstrates the utility of integrating regenerative technologies into the management of severe Morel-Lavallée lesions, especially when traditional skin grafting may be limited by patient factors or wound complexity.

## Conclusions

Morel-Lavallée lesions are frequently underdiagnosed in polytrauma patients, yet their clinical significance cannot be overstated. These shearing injuries disrupt lymphatic and venous structures, creating fluid-filled cavities that often mimic hematomas or abscesses, leading to delayed recognition and management. Our case highlights the critical need for heightened clinical suspicion, as the lesion in our patient was initially missed due to the severity of her traumatic injuries. When finally identified, it had progressed to a necrotic eschar, necessitating an advanced surgical approach incorporating epidermal liquid skin autograft and sclerosing agents to promote healing and prevent recurrence. Given the high morbidity associated with delayed recognition of Morel-Lavallée lesions, we advocate for standardized trauma protocols that incorporate early screening for closed degloving injuries in high-energy mechanisms, particularly in patients with pelvic or lower extremity trauma. This should include routine clinical reassessment of soft tissue compartments during tertiary surveys; a low threshold for advanced imaging when fluctuance, soft tissue swelling out of proportion to exam, or evolving skin changes are present; and early consultation with trauma and reconstructive surgery teams when a lesion is suspected. Additionally, formal documentation prompts within electronic medical records and education of trauma teams on the characteristic imaging and physical exam findings of Morel-Lavallée lesions may reduce missed or delayed diagnoses. Implementing these protocol-driven strategies has the potential to expedite intervention, decrease infectious complications, and improve overall reconstructive outcomes in trauma patients. This case underscores the importance of early diagnosis and intervention in preventing long-term complications and contributes valuable insight into innovative treatment strategies for these often-overlooked soft tissue injuries.
